# Comparative statistical evaluation of greenness, blueness, and whiteness spectrophotometric methods for dexamethasone and chloramphenicol estimation

**DOI:** 10.1038/s41598-025-96091-7

**Published:** 2025-04-21

**Authors:** Heidi R. Abd El-Hadi

**Affiliations:** Faculty of Pharmacy, Pharmaceutical Chemistry Department, Egyptian Russian University, Badr City, Cairo Egypt

**Keywords:** Spectrophotometric methods, Chloramphenicol, Dexamethasone sodium phosphate, Greenness, Whiteness, Blueness, Analytical chemistry, Green chemistry

## Abstract

Five sustainable and validated UV spectrophotometric methods were developed for analyzing chloramphenicol (CHL) and dexamethasone sodium phosphate (DSP) in pure and ophthalmic dosage forms. CHL was detected by zero order spectra method at 292.0 nm in the range 2.00–32.00 µg/mL with limits of detection (LOD) and quantification (LOQ) of 0.96 and 2.88, respectively. DSP was analyzed using the following four techniques: Induce dual wavelength (IDW), fourier self-deconvolution (FSD), ratio difference (RD), and derivative ratio (DD^1^). The IDW method used at 239.0 and 254.0 nm with a linearity range of 4.00–40.00 µg/mL with LOD and LOQ values were 0.93 and 2.79, respectively. The FSD approach used at 242.0 nm, with a linearity range of 2.00–32.00 µg/mL and 0.65, 1.95 as values of LOD and LOQ, respectively. In the linearity range of 4.00–32.00 µg/mL, RD and DD^1^ are applied. RD is utilized at 225.0–240.0 nm, while DD^1^ is carried out at 249.0 nm. Values of LOD and LOQ for RD were 0.70 and 2.10 while for DD^1^ were 0.80 and 2.40, respectively. These methods were evaluated for their environmental sustainability and validated according to ICH guidelines, overcoming challenges like spectral overlap and collinearity. Statistical comparisons with published methods revealed no significant differences.

**Supplementary Information:**

The online version contains supplementary material available at 10.1038/s41598-025-96091-7.

## Introduction

**Fig. 1:**
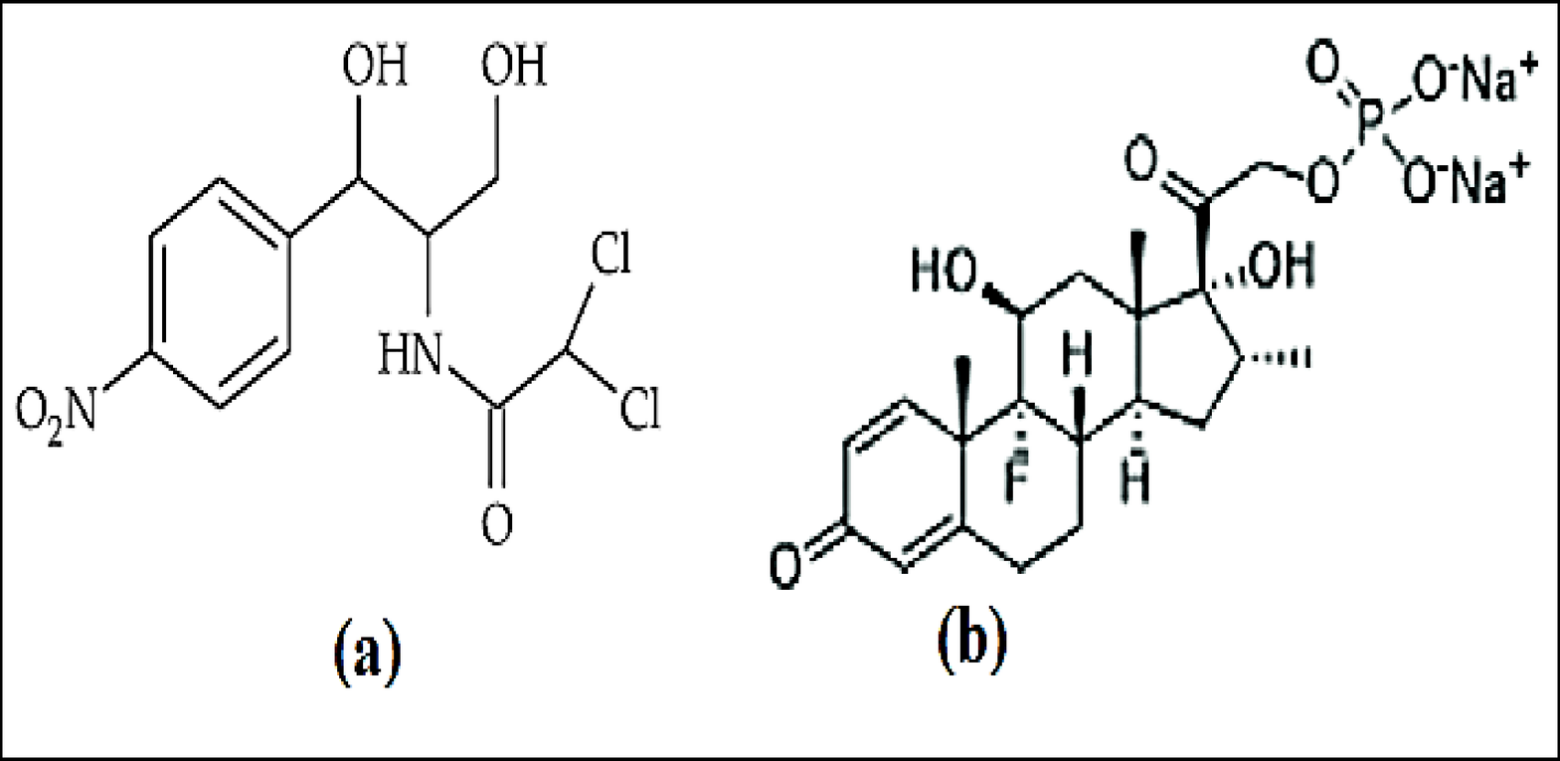
Chemical structure of (**a**) chloramphenicol and (**b**) dexamethasone sodium phosphate.

Chloramphenicol (CHL) is a broad-spectrum antibiotic with antibacterial activity against both Gram-negative and Gram-positive bacteria (Fig. 1a)^1^. Its chemical name is 2,2-dichloro-[1,3-dihydroxy-1-(4-nitrophenyl) propan-2-yl]acetamide^1^. Dexamethasone sodium phosphate (DSP) is an inorganic ester with anti-inflammatory properties (Fig. 1b)^2^. DSP is frequently utilized to treat diseases associated with adrenal cortex insufficiency. The chemical name is 9-fluoro-11b,17,21-trihydroxy-16a-methylpregna-1,4-diene-3,20-dione21-(dihydrogenphosphate) disodium salt^2^. Under trade name Spersadex comp^®^ eye drops, a combination of CHL and DSP are available in the Egyptian market and used for conjunctivitis treatment.

The Analytical eco-scale, Green Analytical Procedure Index (GAPI), and Analytical GREEnness metric (AGREE) are several approaches to evaluate how green a proposed analytical methodology is. The Blue Applicability Grade Index (BAGI) tool allows for a quantitative assessment of the usefulness and practicality of analytical methods (blueness)^3^. Green analytical chemistry is expanded upon by White Analytical Chemistry (WAC), which adds 12 more principles. In addition to the green factors that impact the quality of the method, WAC considers two other significant factors. These elements are the practical (blue) and analytical (red) components^4^.

A review of the literature revealed various chromatographic methods and only one spectrophotometric research for estimating CHL and DSP in pharmaceutical preparations^5–8^. None of the reported methods were assessed based on greenness, whiteness, or blueness.

This study aims to determine CHL and DSP in bulk form and pharmaceutical applications using simple, selective, and eco-friendly spectrophotometric techniques. Additionally, it evaluates greenness, whiteness, and blueness for the first time. The suggested methodologies included Zero order (D^0^), Induce dual wavelength (IDW), fourier self-deconvolution (FSD), ratio difference (RD), and derivative ratio (DD^1^). The developed approaches were statistically compared to a published HPLC method, and no significant difference was found.

## Experimental

### Apparatus

Double-beam JASCO (Tokyo, Japan): An UV-visible spectrophotometer model number V-630 was utilized in conjunction with a software package known as Spectra-II Manager. The width of the spectral slit was 2 nm, and the scan speed was 1000 nm/min.

### Chemicals and reagents

The CHL and DSP used in this experiment were generously provided by Orchidia Co. for Pharmaceutical Ind. (Cairo, Egypt). The official method assessed the purities of CHL and DSP as 99.07 ± 0.72% and 99.36 ± 0.53%, accordingly^9^. All reagents used were analytical grade and did not require any additional purification prior to use. Ethanol was supplied by El-Nasr Pharmaceutical Chemicals Co. (Cairo, Egypt).

### Pharmaceutical Preparation

The Swiss company Novartis Pharma AG produced Spersadex comp ^®^ eye drops. It was bought from the local market in Egypt. It is stated that each 1 mL contains 5 mg of CHL and 1 mg of DSP. The usual dose of eye drops is one drop one to 4 times daily into the lower eyelid.

### Preparation of standard solutions

Separate stock solutions of CHL and DSP (1 mg/mL) were prepared by dissolving 100.0 mg of each drug in 100 mL of ethanol. These solutions were freshly prepared and stored away from light. To prepare the working solutions of CHL and DSP (40.00 µg/mL), transfer 2 mL of each stock solution into two separate 50-mL volumetric flasks, then adjust the volume with ethanol.

### Procedure

#### Construction of zero-order calibration curves

CHL and DSP aliquots were accurately transferred from the working standard solutions (40.00 µg/mL) into 10-mL volumetric flasks. Ethanol was utilized to get the volumes up to the required level. The prepared solutions were scanned between 200.0 and 400.0 nm, and their absorption spectra were recorded for further analysis.

#### Zero order absorption spectra method (D^0^)

CHL can be properly estimated using zero order spectra in which DSP exhibits no absorption. The linearity of CHL was detected at a wavelength of 292.0 nm, and the appropriate CHL concentration and regression equation were calculated.

#### Induce dual wavelength (IDW)

After choosing two wavelengths from the DSP zero-order absorption spectrum (239.0 and 254.0 nm) the absorbance was adjusted by multiplying it by equality factor (F). Plotting the corrected results’ absorbance variation against concentration was done then the regression equation was calculated.

#### Fourier self-deconvolution spectrophotometric method (FSD)

Each drug’s spectrum was evaluated and deconvoluted separately using the FSD function. A calibration curve for DSP in binary mixtures was created using the amplitude of the deconvoluted signal at 242.0 nm. Then, both pharmaceutical formulations and laboratory-prepared mixtures were analyzed using this linear relationship.

#### Ratio difference spectrophotometric method (RD)

DSP was detected using this approach, which involved dividing the recorded zero-order spectra of DSP by that of CHL (4.00 µg/mL). The peak amplitude variation between 225.0 nm and 240.0 nm (ΔP _225.0–240.0 nm_) were plotted against analogue concentrations, and the regression equation was calculated.

#### Derivative ratio spectrophotometric method (DD^1^)

This technique is utilized to calculate DSP. To obtain the first derivative of the stored ratio spectra, we used a scaling factor of 10.0 and Δλ of 4.0 nm. The DD^1^ peak amplitude of the DSP ratio spectra was recorded at 249.0 nm. A regression equation was then calculated by plotting the peak amplitudes at 249.0 nm against the corresponding concentrations on a calibration curve.

#### Analysis of laboratory prepared mixtures

Ethanol was used as a solvent to prepare a set of five different laboratory mixtures of CHL and DSP in different ratios. The mixtures were then measured between 200.0 and 400.0 nm. The aforementioned techniques were used to measure each drug’s concentration independently.

#### Application to pharmaceutical formulation

After carefully transferring 1 mL of Spersadex comp ^®^ eye drops which contains 5 mg of CHL and 1 mg of DSP into 25- mL volumetric flask, the volume was adjusted with ethanol to reach a concentration of 200.0 µg/mL for CHL and 40.0 µg/mL for DSP. After that, 1 mL of the previous solution was transferred into 10 -mL volumetric flasks. The volume was filled with the appropriate solvents to achieve the final concentrations of 4.00 µg/mL of DSP and 20.00 µg/mL of CHL. The concentration of each drug was determined using the corresponding regression equation.

#### Statistical analysis study

The results of the applied methods were compared to those of the reported approach and to each other using a variety of statistical tools, including the Student’s t-test, F-test, one-way ANOVA, normal probability plot, interval plot, boxplot and Tukey’s simultaneous significant difference test^10^.

### Greenness assessment

The ecological impact of the analytical techniques was assessed based on four main criteria: high energy consumption, significant waste generation, excessive chemical use and associated risks, and large quantities of chemicals used^11^. To evaluate the cost-effectiveness and environmental friendliness of the proposed method, three greenness assessment metrics were employed:

#### Analytical eco-scale

The eco-scale tool uses penalty points out of 100 to assess results, with 100 being the ideal score for a green analytical method^12^. Every analytical method parameter, including waste production, energy use, occupational risk, and reagent quantity and quality, is computed and deducted from 100^26^. The approach becomes more economical and ecologically friendly as the score rises. Excellent green analysis is indicated by a score of 75 or higher, acceptable green analysis is represented by a score between 50 and 75, and insufficient green analysis is indicated by a score of 50 or lower^12^.

#### Analytical greenness metric (AGREE)

Using the 12 principles of green analytical chemistry as its evaluation criteria—such as treatment, sample amount and stages, waste, energy consumption, and toxicity—it is an easy-to-use, comprehensive tool to assess environmental friendliness of analytical method^13^. A single 0–1 scale is created from each of these variables. The process and weight of each principle are indicated by the width of the segment to which it is related and by the color scale of the resulting pictogram. The assessed techniques are demonstrated to be more ecologically friendly in the central region of the pictogram, with values close to 1 and a dark-green background^13^.

#### Green analytical procedure index (GAPI)

This tool provides detailed information on fifteen aspects of the analytical technique, each represented by five pentagrams, with each pentagram representing a step in the analytical procedure. These steps are sampling, preparation, instrumentation, the solvents and reagents used, and the overall objective of the analytical method^14^. According to the GAPI color scheme, green indicates greater ecological tolerance, yellow indicates less ecological tolerance, and red indicates a significant environmental risk^25^.

### Blue applicability grade index (BAGI)

Practicability of analytical chemistry method is a metric tool that assigns a score between 25 and 100; the higher the score, the more practical the method^3^. Ten primary attributes that are evaluated by this tool are type of analysis, the number of analytes that are determined simultaneously, the number of samples that can be analyzed in an hour, the type of reagents and materials used in the analytical method. In addition to the necessary instrumentation, the number of samples that can be treated simultaneously, the need for preconcentration, the degree of automation, the type of sample preparation, and the amount of sample^15^.

### Whiteness assessment

There are four red, four green, and four blue principles among the twelve WAC principles^4^ which called the RGB12 model. The green ones represent green chemistry (toxicity of reagents, number of reagents and amount of waste, energy), the blue ones represent the practical side (cost-efficiency, time-efficiency, requirements and operational simplicity). While the red ones represent analytical performance (scope of application, LOD and LOQ, precision and accuracy). Whiteness scores were computed for proposed methods. A score of 100 indicates that the method is well-suited for a planned application in terms of principle, while a score of zero indicates the worst outcome^16^.

## Result and discussion

Spectrophotometric methods are quick, simple, and affordable when compared to other approaches that call for sophisticated equipment or chemical pretreatment, like chromatographic techniques. According to the literature review, there was only one spectrophotometric research for the determination of our binary mixture. Five, simple and eco-friendly UV spectrophotometric methods for concurrently evaluating CHL and DSP in pharmaceutical and bulk forms are provided by the current study.

### Method development

#### Zero order absorption spectra method (D^0^)

**Fig. 2:**
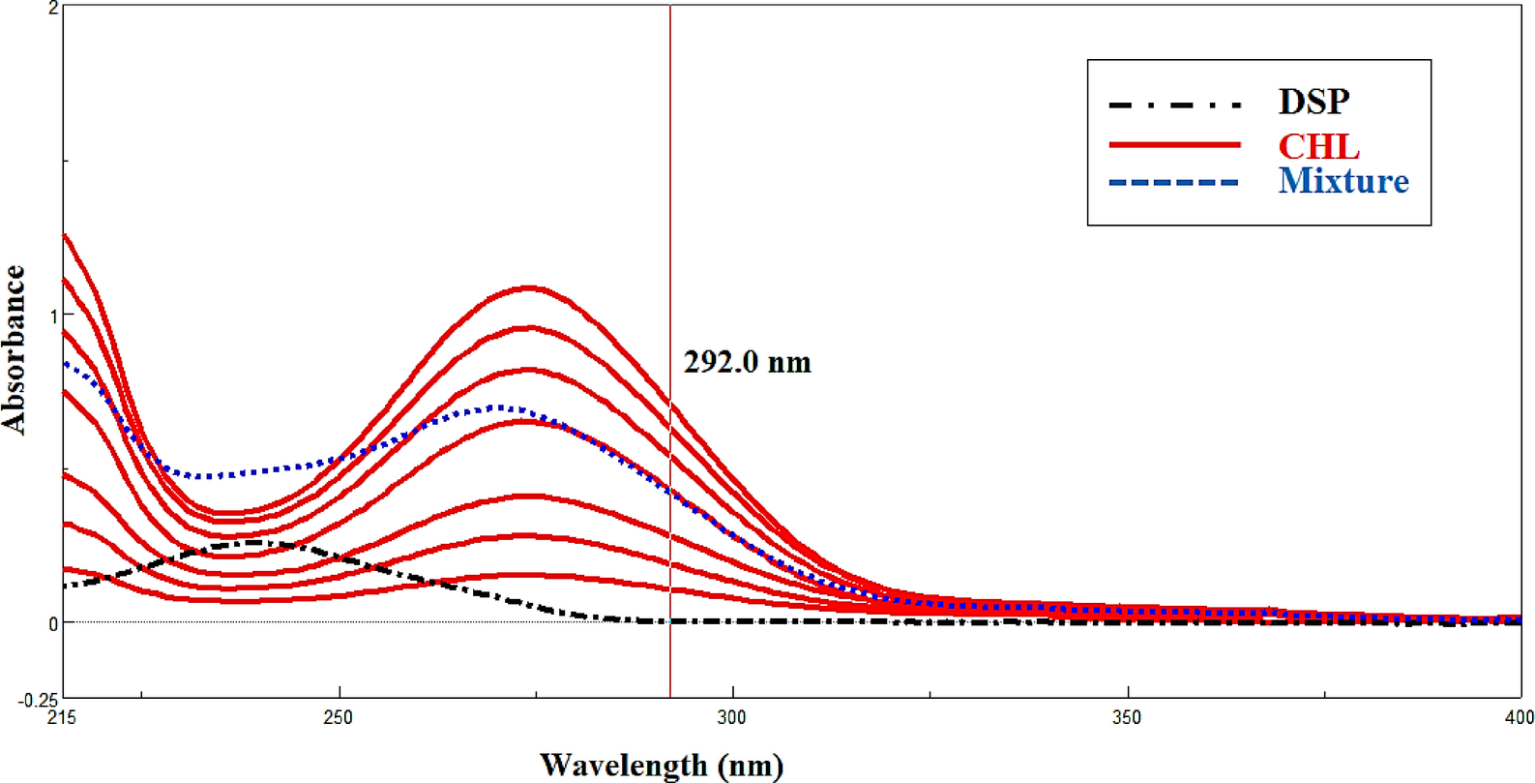
Zero order absorption spectra of 4.00–32.00 μg/mL CHL and 10.00 μg/mL DSP and lab mixture of 18.00 μg/mL CHL and 10.00 μg/mL DSP using ethanol as a blank. CHL absorbance was measured at 292.0 nm while DSP showed no absorbance.

It is the most straightforward method of assessing a mixture because only one ingredient can be directly detected, and the other drug don’t interfere with it^17^. Figure 2 represent to zero order absorption spectra of (4.00–32.00 µg/mL) CHL and 10.0 µg/mL DSP using ethanol as a blank. CHL absorbance was measured at 292.0 nm while DSP showed no absorbance.

#### Induce dual wavelength (IDW)

**Fig. 3:**
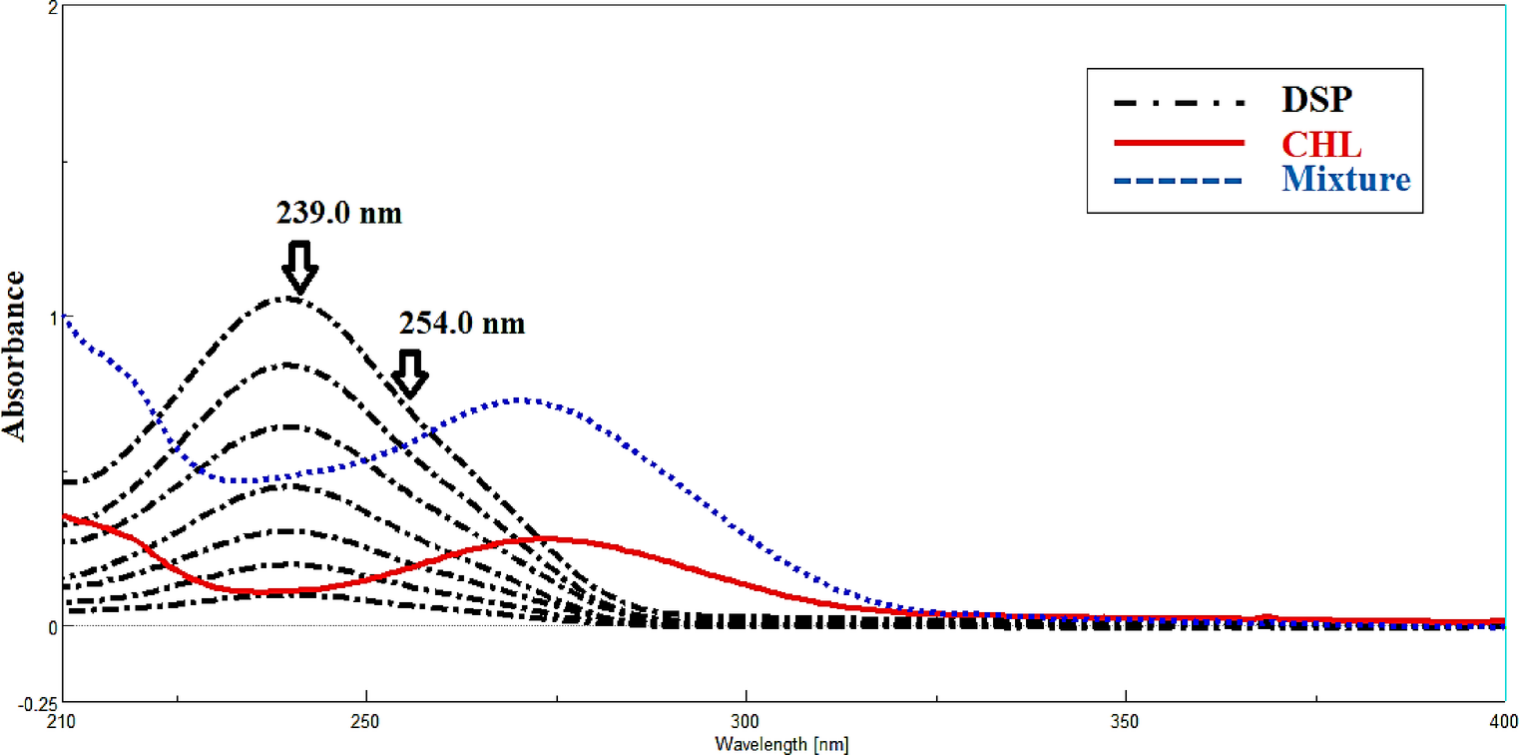
Zero order absorption spectra of 4.00–40.00 µg/µL DSP, 8.00 µg/µL of CHL and and lab mixture of 18.00 µg/mL CHL and 10.00 µg/mL DSP using ethanol as a blank. DSP was measured at 239.0 nm and 254.0 nm using induce dual wavelength method.

Recently, two components, X and Y, with fully overlapping spectra in a binary mixture were resolved and estimated using the IDW. Any two wavelengths (λ1 and λ2) can be used to apply IDW without any limitations^18^. Two wavelengths on the zero-order spectrum of DSP where the difference in absorbance for CHL does not equal zero were chosen for our study in order to determine DSP in the presence of CHL. We multiplied by the equality factor (F) to get the absorbance difference for CHL to be zero, but this difference was still significant for DSP to remove the interference from CHL. By dividing the absorbance at λ239.0 by the absorbance at λ254.0, the equality factor (F) was determined from the zero-order absorption spectrum of DSP and was 0.617. In the linear range of (4.00–40.00 µg/mL), the absorbance difference of DSP as a function of concentration was plotted to create calibration graphs, as seen in (Fig. 3).

#### Fourier self‑deconvolution spectrophotometric method (FSD)

**Fig. 4:**
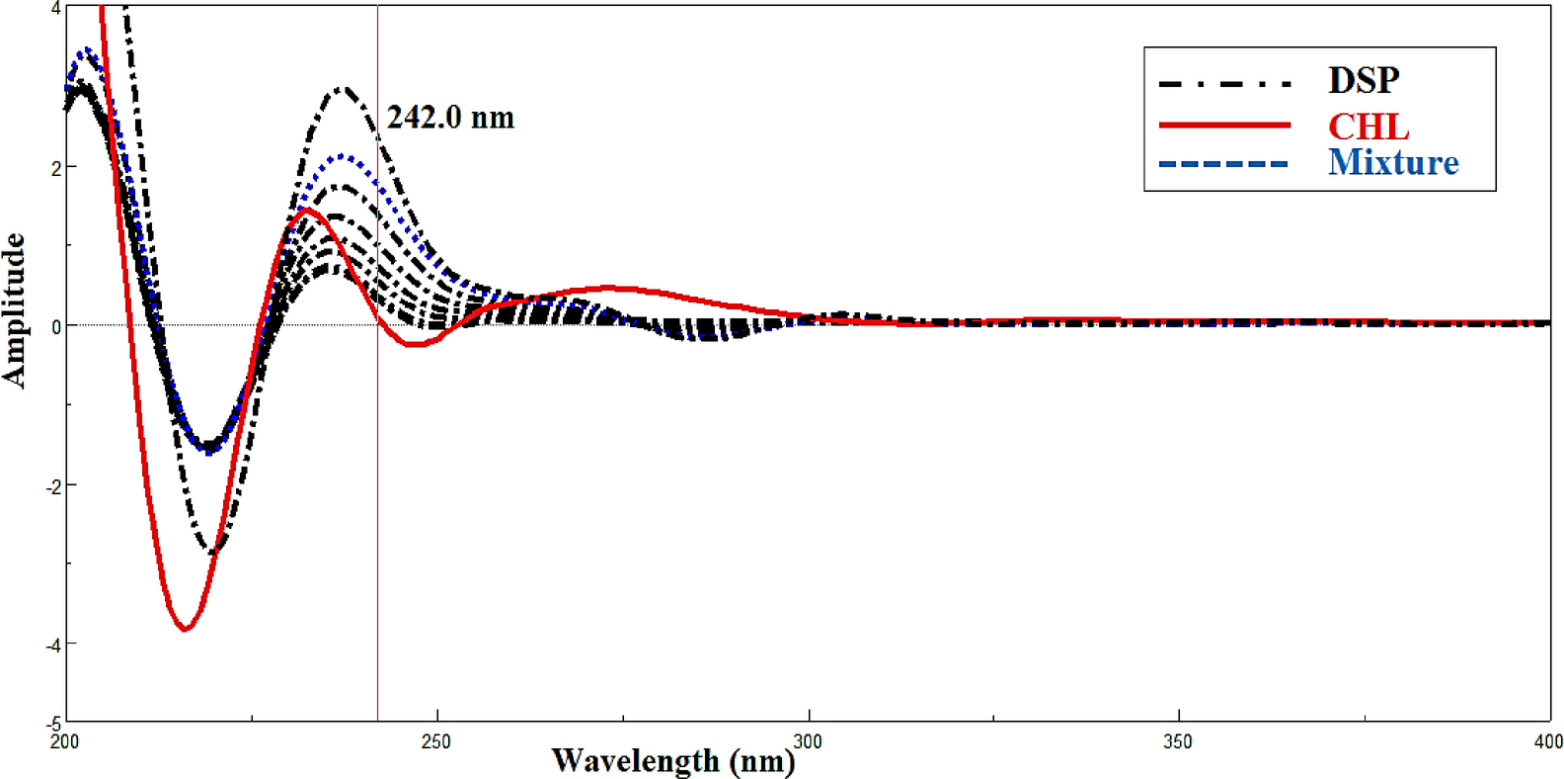
Deconvoluted spectra of DSP (2.0–32.0 µg/mL) and lab mixture of 18.00 µg/mL CHL and 10.00 µg/mL DSP which determined at 242.0 nm, zero-crossing point of CHL using ethanol as a solvent.

The existing approach relies on locating zero-crossing points or regions of no contribution in the mixed spectra, excluding physical convolution brought on by instrumental distortions. Reversing the spectrophotometer’s distorting effects on the recorded spectrum is the fundamental concept of FSD^19^. With the zero-order spectrum for CHL already determined, this approach was used to resolve and assess the binary mixture of CHL and DSP. With Full Width at Half Maximum (FWHM) function value of 65. In the linear range of (2.00–32.00 µg/mL), a calibration curve for DSP was made at 242.00 nm, which corresponds to the zero-crossing point for CHL as illustrated in (Fig. 4).

#### Ratio difference spectrophotometric method (RD)

**Fig. 5:**
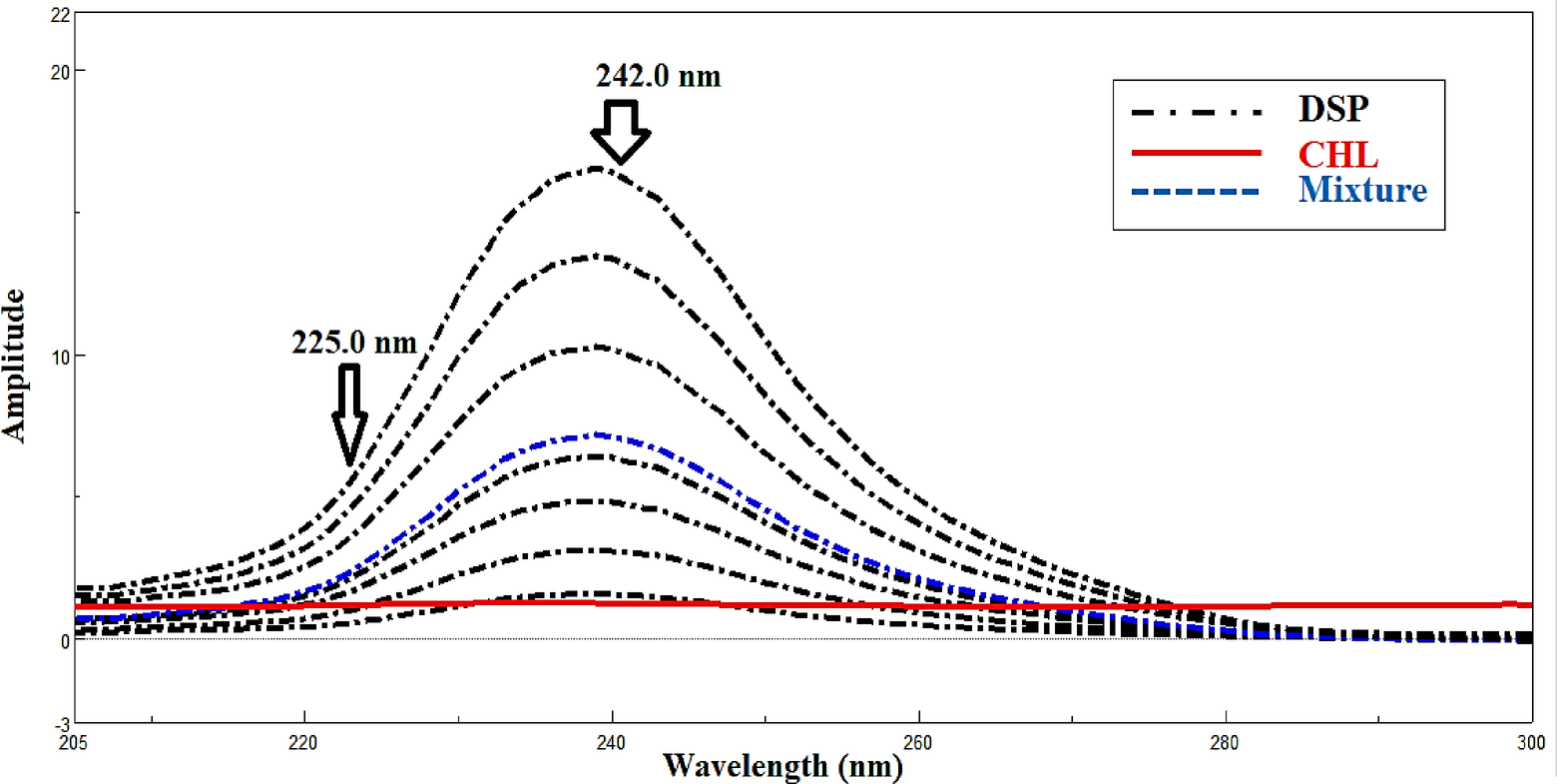
Ratio difference spectra of 4.00–32.00 µg/mL DSP and lab mixture of 18.00 µg/mL CHL and 10.00 µg/mL DSP using 4.00 µg/mL CHL as a divisor. DSP was detected by obtaining the amplitude differences (ΔP) between 242.0 and 225.0 nm where CHL showed no ΔP between the two selected points.

This approach can effectively handle binary mixtures, only taking into account the two component spectra at specific wavelengths^17,20^. The process consists of two key steps: first, choose an appropriate divisor concentration with low noise and high sensitivity. The most sensitive CHL divisor concentration was found to be 4.00 µg/mL. The second step is to select a pair of wavelengths with good linearity. The best results were obtained between 225.0 and 240.0 nm. The amplitude differences (ΔP) between these wavelengths were measured after dividing the stored zero spectra of DSP by 4.00 µg/mL of CHL (Fig. 5). While CHL plateaued, DSP showed significant amplitude variations at specific wavelengths within a linearity range of 2.00–32.00 µg/mL.

#### Derivative ratio spectrophotometric method (DD^1^)

**Fig. 6:**
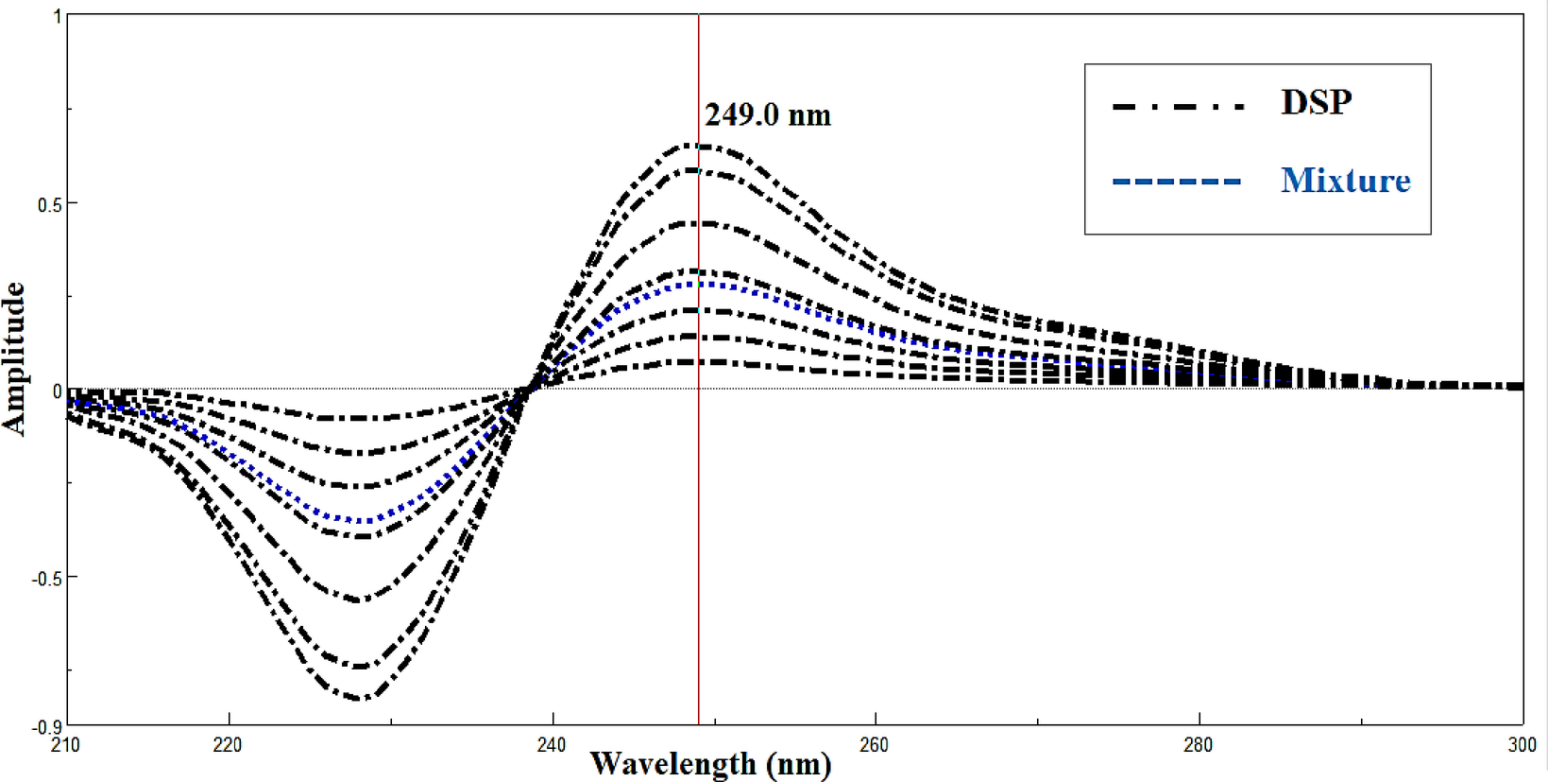
First derivative ratio spectra of 4.00–32.00 µg/mL DSP and lab mixture of 18.00 µg/mL CHL and 10.00 µg/mL DSP using 4.00 µg/mL of CHL as a divisor. DSP was detected at its maximum wavelength 249.0 nm.

The primary advantage of this approach is its ability to exclude the entire spectrum of the interfering substance, allowing for calibration at each wavelength. This allows for simple measurements at the minimum or maximum wavelengths while maintaining optimal sensitivity^17,21^. The optimal conditions for obtaining the first derivative of the stored ratio spectra of DSP, with a linearity range of 4.00–32.00 µg/mL, were detected at its maximum wavelength of 249.0 nm. Concentration 4.00 µg/mL of CHL was used as a divisor which show low noise and high sensitivity, as shown in (Fig. 6).

### Method validation

According to the ICH guidelines, which include linearity, range, accuracy, and precision, the introduced strategies were validated^22^.

#### Linearity and range

According to the ICH guidelines, linearity should be established with at least five concentrations. To ensure linearity, calibration graphs for each method were plotted, connecting the response to the corresponding CHL and DSP concentrations. A summary of the findings is presented in (Table 1).

**Table 1:** Regression and validation parameters of the established spectrophotometric methods for determination of CHL and DSP.

Validation parameters	CHL	DSP			
	D^0^ (292.0 nm)	IDW (239.0–254.0 nm)	FSD (242.0 nm)	RD (ΔP_225.0–240.0 nm_)	DD^1^ (249.0 nm)
Linearity range (µg/mL)	4.00–32.00	4.00–40.00	2.00–32.00	4.00–32.00	
Correlation coefficient (r)	0.9994	0.09996	0.9996	0.9996	0.9995
Slope	0.018742	0.015108	0.046241	0.236233	0.018483
Intercept	0.017978	− 0.01055	0.169126	− 0.15879	− 0.0113
Accuracy^a^ (mean% ± SD)	100.12 ± 0.72	100.36 ± 1.17	100.31 ± 1.77	100.33 ± 1.37	100.80 ± 0.81
Precision^b^					
Repeatability	1.08	0.79	1.10	1.16	0.97
Intermediate	1.12	0.94	1.07	1.25	1.29
LOD^c^	0.96	0.93	0.65	0.70	0.80
LOQ^c^	2.88	2.79	1.95	2.10	2.40

^a^Average of three different concentrations repeated three times within the day. ^b^Precision was e valuated by measuring the response of three concentrations of each drug three separate times on the same day (repeatability) and on three different days (intermediate precision).^c^LOD and LOQ were calculated from the standard deviation (s) of the response and the slope of the calibration curve (S) according to the following equations: LOD = 3.3(s/S) and LOQ = 10(s/S).

#### Accuracy

As shown in (Table 1), it is represented as the means of percentage recoveries and standard deviations (Mean ± SD) of three different concentrations. The accuracy results for the proposed method were satisfactory.

#### Precision

*Repeatability:* Using the previously described procedure under linearity, three concentration levels (10.00,18.00,30.00 µg/mL) for both CHL and DSP were analyzed in triplicates intra-daily. The results are displayed in (Table 1).

*Intermediate:* The aforementioned CHL and DSP samples under repeatability were analyzed in triplicates on three consecutive days using the procedures stated under linearity. The results are displayed in (Table 1).

#### Limit of detection (LOD) and limit of quantification (LOQ)

They were computed using the following formulas: LOD = 3.3 (s/S) and LOQ = 10 (s/S) based on the response’s standard deviation (s) and the calibration curve’s slope (S). The LOD and LOQ values were displayed in (Table 1).

#### Specificity

Through the examination of laboratory mixtures containing different ratios of CHL and DSP, the specificity of the established techniques was estimated. The methods’ high specificity was demonstrated by the positive outcomes, as shown in (Table 2).

**Table 2:** Determination of CHL and DSP in their laboratory prepared mixtures by the proposed spectrophotometric methods.

Claimed concentration taken (µg/ mL)		CHL	DSP			
CHL	DSP	D^0^ Recovery (%)	IDW Recovery (%)	FSD Recovery (%)	RD Recovery (%)	DD^1^ Recovery (%)
12.00	32.00	100.65	99.84	101.92	99.26	100.66
18.00	10.00	99.96	98.80	98.39	99.37	98.06
30.00	6.00*	100.93	100.55	100.90	100.59	100.03
6.00	12.00	98.72	98.13	100.81	98.48	101.00
20.00	10.00	101.52	99.53	101.48	101.18	99.26
Mean (%)		100.35	99.37	100.70	99.78	99.80
SD		0.95	0.83	1.22	0.97	1.05

*The same ratio in dosage form.

### Application of dosage form and statistical study

The results of established methods that applied for dosage form are shown in (Table 3). The results of the applied methods were compared to those of the published approach as well as among themselves using a variety of statistical tools. Student’s t-test and F-test were used to compare the proposed and reported methods, and no significant difference was found (Table 4).

**Table 3:** Determination of CHL and DSP in a pharmaceutical formulation using the proposed methods.

Spersadex comp ^®^ eye drops ( each 1 mL contains 5 mg of CHL/1 mg of DSP)		Amount added of standard (µg/ mL)	Apparent recovery % ± SD ^a^
CHL	D^0^	20.00	98.60 ± 0.52
DSP	IDW	4.00	99.84 ± 1.12
	FSD		99.80 ± 0.69
	RD		100.10 ± 0.80
	DD^1^		99.53 ± 0.94

^a^Average of three determinations.

**Table 4:** Statistical analysis of the results obtained by the developed methods and the reported HPLC methods for the determination of CHL and DSP in pharmaceutical preparation.

Parameters	CHL		DSP				
	Proposed methods	Reported method	Proposed methods				Reported method
	D^0^	HPLC^a^	IDW	FSD	RD	DD^1^	HPLC^a^
Mean^b^ (%)	98.60	99.56	99.84	99.81	99.54	100.10	100.33
SD	0.52	0.46	1.12	0.69	0.80	0.94	0.37
Variance	0.27	0.21	1.26	0.48	0.64	0.89	0.14
n	3	3	3	3	3	3	3
Student’s *t*-teste (2.78)^c^	2.36	–	0.71	1.15	1.55	0.39	–
*F*-value (19.00)^c^	1.26	–	8.88	3.38	4.53	6.27	–

^a^HPLC method: phosphate buffer and methanol (50:50% v/v) at flow rate of 1.2 mL/min, detection at 254.0 nm^5^. ^b^Average of 3 experiments. ^c^Figures between parentheses represent the corresponding tabulated values of t and F at *P* = 0.05.

A one-way ANOVA test was additionally performed to compare the developed approaches (see Table 5), and the results showed no significant difference between the groups, with calculated F-values lower than the critical value.

**Table 5:** One-way ANOVA results for determination of proposed and reported methods of DSP in pharmaceutical formulation.

Source of variation	SS	df	MS	F	*P*-value	F crit
Between Groups	1.41	4	0.35	0.49	0.74	3.47
Within groups	7.20	10	0.72	–	–	–

**Fig. 7:**
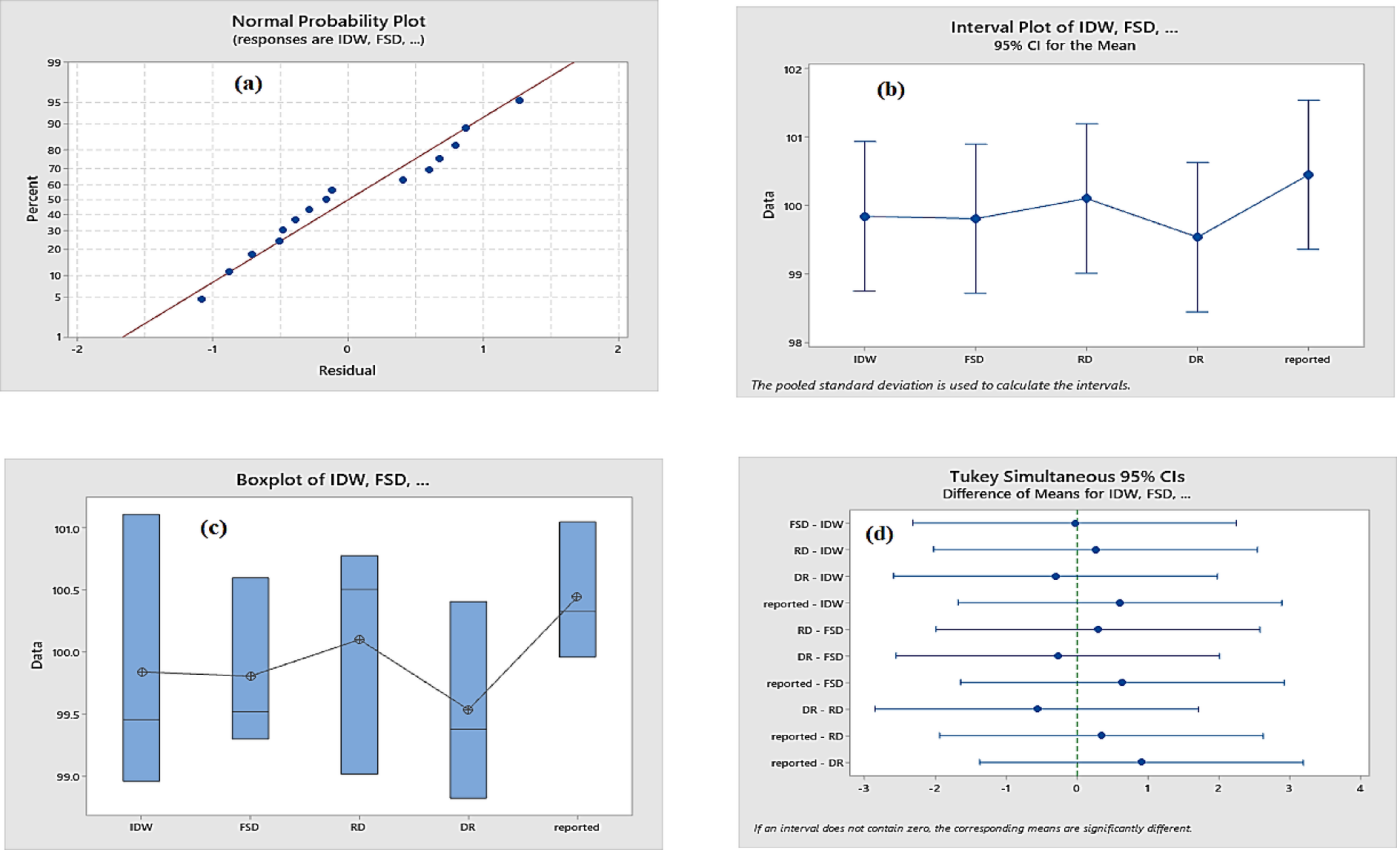
(**a**) normal probability plot (**b**) Interval plot, (**c**) boxplot, and (**d**) Tukey’s simultaneous significant difference test for the established and reported methods of DSP in pharmaceutical dosage form.

The normal probability plot is an additional technique for assessing whether data are normally distributed (Fig. 7a). In different pharmaceutical formulations, the data satisfy the normal distribution if the straight line crosses most of the data points^10^.

The interval plot test was the third statistical tool utilized^23^. Plots of confidence intervals display them as vertical lines with the center point of the interval mean. Assume that the diagram’s data group intervals for each strategy overlap. These plots show that there are no appreciable differences between reported and offered approaches in different pharmaceutical formulations (Fig. 7b).

The boxplot, which displays the distribution of data among groups, is another essential data visualization tool^23^. Boxplots of suggested and documented methods in a range of pharmaceutical formulations are shown in (Fig. 7c). The middle quartile is represented by the central box, which has a line showing the data median, upper lines showing higher values, and whiskers showing lower values. The data distribution within each data category is shown in the boxplot.

The Tukey’s simultaneous significant difference test is the final statistical tool^24^. It is a useful tool for determining whether the mean values of the different groups differ from one another. Each group’s data interval is displayed as a horizontal line in (Fig. 7d), with the mean value for each group in the center. The overlap of the intervals showed that the mean values of the reported and suggested methods in different pharmaceutical formulations did not differ significantly.

### Greenness assessment

#### Analytical eco‑scale

The suggested approaches yield 7 penalty points with an analytical eco-scale score equal to 93 while the reported method has 10 penalty points with an analytical eco-scale score equal to 90, as shown in (Table 6), indicating established methods greener than reported one.

**Table 6:** Penalty points (PPs) for the proposed and reported methods. Analytical eco-scale score = 100 (the ideal score of green analytical method).

Parameters	Established methods	Reported method
Reagents		
Ethanol	4	–
Methanol	–	6
Phosphate buffer	–	0
Instrument		
Energy (> 0.1kWh per sample)	0	1
Occupational hazard	0	0
Waste	3	3
Total PPs	Ʃ7	Ʃ10
Analytical Eco-scale score	93 Excellent green analysis	90 Excellent green analysis

Analytical eco-scale score > 75 (excellent green analysis). Analytical eco-scale score 50–75 (the green analysis is acceptable). Analytical eco-scale score < 50 (the green analysis is inadequate).

#### Analytical greenness metric (AGREE)

**Fig. 8:**
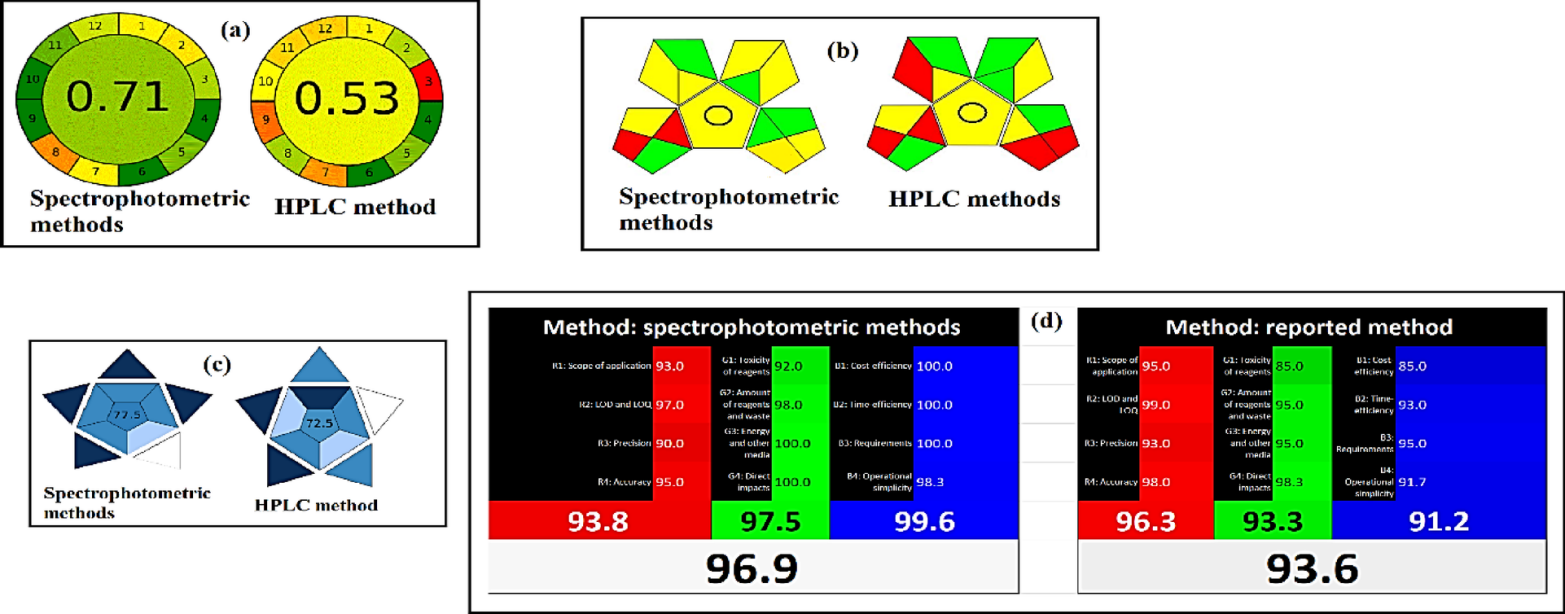
(**a**) AGREE assessment of the green profile (**b**) GAPI pictograms (**c**) BAGI tool (**d**) RGB12 algorithm for the suggested and reported approaches.

In (Fig. 8a), the 12 input variables are shown as colored pictograms along which were calculated for recommended and reported approaches. AGREE score for the established methods was 0.71 while was 0.53 for reported one. The developed approach is therefore more environmentally friendly than the reported HPLC approach, as determined by the AGREE score.

#### Green analytical procedure index (GAPI)

To determine whether an analytical method followed the principles of green chemistry, its environmental impact had to be assessed. As illustrated in (Fig. 8b), established methods received two red, five green, and eight yellow ratings while reported one has five red, five green, and five yellow ratings. So, the established methods were greener than the reported one. The GAPI tool assessed these analytical processes and assigned them to a green assessment profile. This profile describes how the approved method is user-friendly, environmentally safe, and can be used for both measurement and characterization without the need for extraction methods. Furthermore, it provided a simple procedure for reducing waste and the use of hazardous materials.

### Blue applicability grade index (BAGI)

In (Fig. 8c), ten primary parameters were calculated for established and reported approaches and the outcomes showing equal scores to 77.5 and 72.5, respectively. that demonstrates the superior functioning and applicability of our suggested approach.

### Whiteness assessment

According to an analysis of whiteness using the RGB12 model, the coherence and synergy of the analytical, ecological, and practical features are revealed when red, green, and blue light beams combine to create the illusion of whiteness^15^. Whiteness scores were calculated for the proposed and reported methods using the red, green, and blue principles. Tabular data of each criterion used in RGB model assessment was shown in (Table S1). Fig. 8d displays these findings, demonstrating the superiority of the recommended strategy from a whiteness perspective. Score of whiteness for recommended methods was 96.9 while for HPLC method was 93.6.

## Conclusion

The challenging binary mixture of CHL and DSP was analyzed for the first time without prior separation using a combination of five spectrophotometric techniques. These methods included the derivative ratio, ratio difference, fourier self-deconvolution, Induce dual wavelength, and zero order absorption spectra. These approaches proved to be quick, easy, sensitive, and precise to assess CHL and DSP in both pharmaceutical and pure forms. The methods utilized were validated according to ICH guidelines and produced highly accurate and precise results for CHL and DSP determinations. The proposed approaches to drug identification are more practical and less complex than the HPLC method. Additionally, the proposed methods showed better performance with different scores on a range of eco-scale tools, such as RGB12, AGREE, GAPI, and BAGI when compared with reported one.

## Electronic supplementary material

Below is the link to the electronic supplementary material.

Supplementary Material 1

## Data Availability

The datasets used and/or analyzed during the current study are available from the corresponding author on reasonable request.
