# Supplementary figures and images for: Comparative statistical evaluation of greenness, blueness, and whiteness spectrophotometric methods for dexamethasone and chloramphenicol estimation
Source: Sci Rep. 2025 Apr 21;15:13772. doi: 10.1038/s41598-025-96091-7 (PMC12012086; doi:10.1038/s41598-025-96091-7)

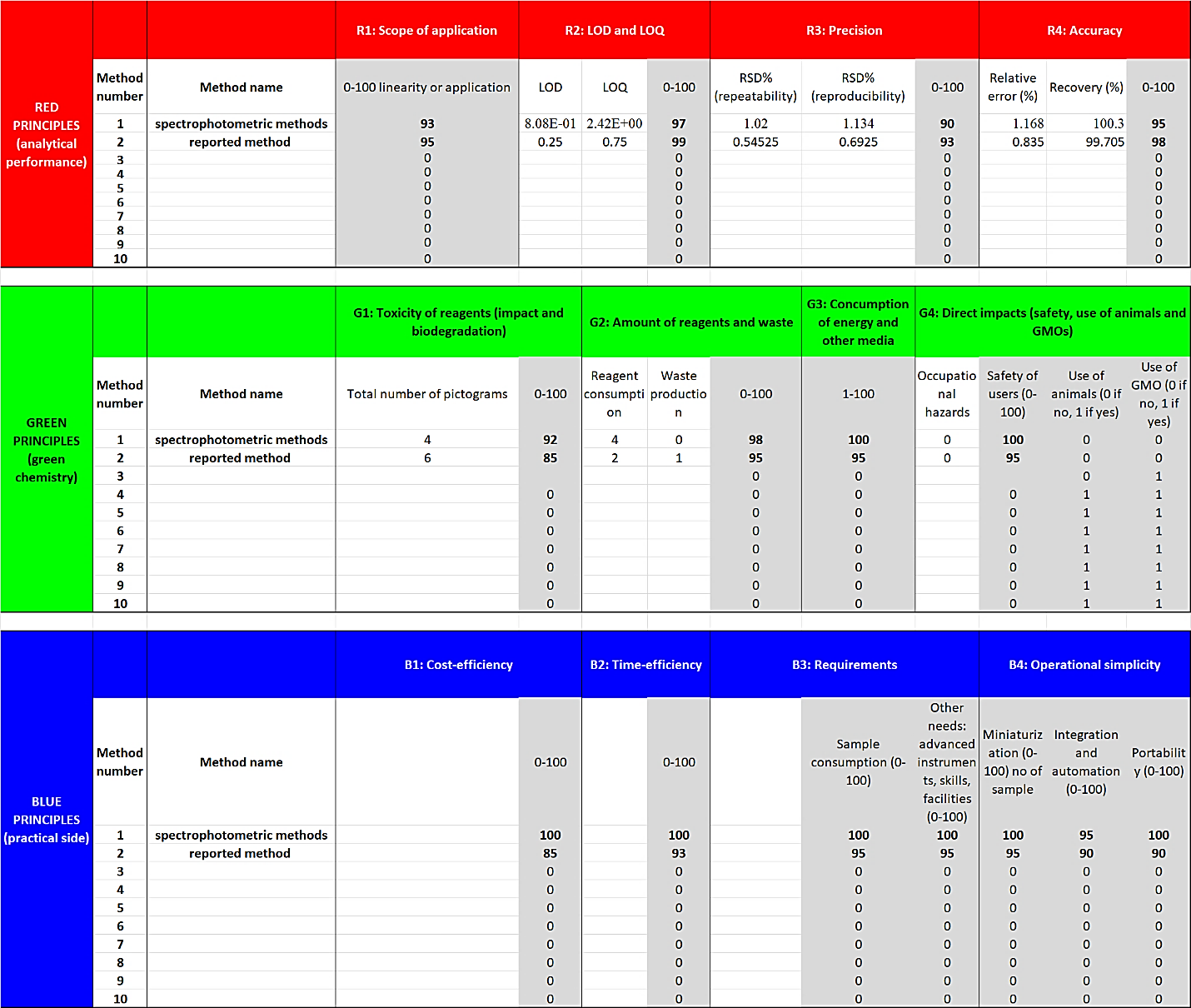


**Table S1:** Tabular data of each criterion used in RGB model assessment.

Supplement: Supplementary file 1 — Supplementary Material 1 [file 41598_2025_96091_MOESM1_ESM.docx]
